# Multilayer PEO coatings with encapsulated cerium for active corrosion protection of aluminium

**DOI:** 10.1038/s41529-025-00560-3

**Published:** 2025-03-07

**Authors:** Safiya Al Abri, Tess Knowles, Yitao Pan, Aleksey Yerokhin, Beatriz Mingo

**Affiliations:** 1 Department of Materials, Henry Royce Institute, The University of Manchester, Oxford Rd, Manchester, M13 9PL UK; 2 Department of Applied Sciences, Applied Chemistry Section, University of Technology and Applied Sciences, Al Khuwair, Muscat, P.O. Box 74 Sultanate of Oman

**Keywords:** Metals and alloys, Ceramics, Nanoparticles

## Abstract

This work aims to develop multilayer coating systems to enhance the long-term corrosion performance of aluminium-based components. The systems consists of a high-performance ceramic matrix that provides physical barrier protection, and a topcoat layer containing encapsulated Ce-based inhibitors, offering active corrosion protection through controlled released mechanisms. Two types of nanoparticles were used for the encapsulation, zeolite and halloysite nanotubes, each with different release triggers and kinetics. Multifunctional coatings demonstrated a superior corrosion performance compared to the passive unmodified coatings. Inhibitor release from the nanoparticles was triggered by ionic exchange processes and changes in pH associated with corrosion activity.

## Introduction

Plasma electrolytic oxidation (PEO) is a versatile surface coating technology used on valve metals (aluminium, titanium, magnesium, etc.) to enhance surface properties such as corrosion, wear, thermal resistance, etc^1^. PEO is an electrolytic technique where the coating grows through plasma-assisted oxidation of the metallic substrate and by the incorporation of elements from the electrolyte. This results in ceramic crystalline coatings with excellent performance, considerably better compared to those formed by anodising, which is possibly the most widely used electrolytic method in industry for improving the surface properties of aluminium. Moreover, PEO employs environmentally friendly electrolytes, requires no stringent substrate pre-treatments, and most importantly, allows the coating of components with complex shapes^2,3^.

The morphology of PEO coatings is characterised by two layers: an inner layer adherent to the substrate, which provides passive protection against corrosion, and an outer porous layer that provides excellent anchorage for subsequent sealing topcoats. Sealing post-treatments are applied with a wide range of purposes; they have been used to improve the barrier capacity of the coating, therefore increasing its corrosion resistance^4^; to improve the wear performance^5^; or to incorporate functionality, such as superhydrophobicity^6^, antibacterial properties^7^, or photocatalytic properties, amongst others. Sealings for corrosion protection are commonly performed in boiling water^8^, in solutions containing corrosion inhibitors^4^, or by using polymeric or sol-gel top-coats^9^. Although sealings can considerably improve corrosion resistance^10^, after prolonged exposure times, the barrier layer tends to fail, compromising the performance of the material. In previous works by the authors^11^ it was revealed that incorporating encapsulated corrosion inhibitors was a promising strategy to provide extended corrosion protection even after coating failure. Encapsulation is key to provide a supply of corrosion inhibitors on demand, when corrosion initiation takes place since free inhibitors are usually leached out of the coating even before corrosion phenomena occur, limiting their inhibition effect.

This work aims to functionalise alumina coatings using two types of nanoparticles, zeolite and halloysite nanotubes (HNT) to encapsulate Ce^3+^, an effective corrosion inhibitor for aluminium. The selection was made based on their availability, size (they should be small enough to fit within the coating’s porosity), loading capacity, and release trigger, which is directly related to the electrostatic interactions between the nanoparticle and the inhibitor.

Zeolite nanoparticles are composed of hydrated aluminosilicates organised in a 3-dimensional framework and connected by oxygen atoms located at the corners of its tetrahedral structure^12^. This configuration forms channels and cages (super-cages and solidate cages) with uniform pore sizes that can selectively be occupied with various molecules and ions. Al^3+^ ions in its structure partially substitute Si^4+^, leading to an overall negatively charged configuration, which is counterbalanced by cations, often alkali and alkaline earth metal ions located in the porosity^13,14^. These cations can be easily substituted with other cations by ionic exchange. The cation’s residence in the zeolite pores does not alter or modify the structure as they are weekly bonded electrostatically. Zeolite is available naturally from minerals and can be synthesised from kaolinite or fly ash^15^. The synthesis of zeolite is costly; however, the ability to synthesise it from flay ash waste is an advantage in mitigating the environmental consequences associated with landfill disposal^16^. HNT is more cost-effective and is readily available commercially. It is a natural clay mineral composed of two layers of aluminosilicates (Al_2_Si_2_O_5_(OH)_4_·*n*H2O) in a 1:1 ratio and has a tubular structure with hollow interiors^17^. The *n* water molecules in the chemical formula usually range between 0 and 2 and are usually located in the interlayer spacing^18^. The length of HNT ranges between 200 and 2000 nm, with an exterior diameter of 50–100 nm and an interior lumen diameter of 10–20 nm^19,20^. The inner lumen of HNT consists of alumina or aluminium hydroxide, while the exterior surface consists of silica, siloxane, or silicon hydroxide^20–23^, depending on the pH. In the pH range of 3–8.5, the interior becomes positively charged, and the external surface is negatively charged. This charge distribution enables the selective encapsulation of negatively charged species within the inner layers and the immobilisation of positively charged species on the exterior surface, attracted electrostatically^21,22,24^.

This work focuses on developing multilayer systems with dual functionality to increase the long-term corrosion performance of A1050 aluminium alloy. The ceramic PEO coating provides physical barrier protection to the substrate, while the top-coat layer containing encapsulated corrosion inhibitors offers active corrosion protection through controlled released mechanisms. The inhibitor-nanocontainer systems are thoroughly characterised with a strong emphasis on correlating the release triggers and kinetics with the corrosion initiation process. The multilayer coatings are evaluated using electrochemical corrosion testing, and the corrosion inhibition mechanism is established considering environmental factors (i.e. pH and presence of Na^+^ and Cl^-^ions), inhibitor release triggers, kinetics and corrosion inhibition effect on the aluminium substrate.

## Methods

### Materials

PEO process was performed on A1050 aluminium alloy (19 × 19 × 6 mm) with a total surface area of 11.7 cm^2^. The chemical composition of A1050 in weight percent (wt%) is 0.25 Si, 0.4 Fe, 0.05 Cu, 0.05 Mg, 0.07 Zn, 0.05 Ti, and 99.5 Al. Prior to the PEO process, the aluminium alloy was ground to a grit size of 1200 and washed with deionised water, followed by drying in a warm air stream.

The chemicals used in this study include sodium chloride (NaCl), sodium silicate (Na_2_SiO_3_), potassium hydroxide (KOH), sodium hydroxide (NaOH), hydrochloric acid (HCl), and snakeskin tube (3.5 K molecular weight cut-off, 22 mm diameter) purchased from Fisher Scientific, halloysite nanotubes and cerium (III) nitrate hexahydrate (Ce(NO_3_)_3_·6H_2_O) purchased from Sigma–Aldrich, and zeolite Y sodium purchased from Alfa Aesar (5.1:1 mole ratio SiO_2_:Al_2_O_3_, 900 m^2^/g).

### Encapsulation of Ce^3+^ into zeolite

Ce^3+^ ions were incorporated into the porosity of the zeolite nanocontainers using the immersion approach. 20 g l^−1^ of zeolite was added to a solution containing 0.2 M of Ce(NO_3_)_3_·6H_2_O. Thereafter, the mixture was stirred for 24 h at room temperature, followed by centrifugation to isolate zeolite nanocontainers, and dried at 80 °C for 15 h. Hereafter, the Ce^3+^ ions encapsulated in zeolite are denoted as Ce-Zeolite.

### Encapsulation of Ce^3+^ into halloysite nanotubes

The Ce^3+^ was loaded into HNT by vacuum-induced capillarity^11^. In brief, 20 gl^−1^ of HNT was suspended in 0.2 M Ce(NO_3_)_3_·6H_2_O solution at room temperature and placed in a desiccator. The air in the desiccator was evacuated by vacuum pumping and maintained under vacuum for 10 min. Afterwards, the vacuum pump was closed, and the suspension was kept under vacuum for 60 min. Subsequently, the desiccator was subjected to air, and the process was repeated three times to maximise the loading of Ce^3+^ and prevent its accumulation at the end of HNT nanotubes. The suspension was then centrifuged, and the solid residue was collected and dried at 55 °C for 24 h. Thereafter, Ce^3+^ loaded in HNT is referred to as Ce-HNT.

### Release studies

The release of Ce^3+^ from the nanocontainers was investigated using a snakeskin tube, which retains the molecules based on their molecular weight. Two hundred milligrams of the nanocontainers were dispersed in a 5 ml solution of 3.5 wt% NaCl and then transferred inside the snakeskin tube. Subsequently, the snakeskin tube is immersed in 95 ml of 3.5 wt% NaCl solution. This only allows the release of Ce^3+^ from the nanocontainers to the NaCl solution while retaining the nanocontainers inside the tube. At a predetermined time interval, 5 ml of solution was taken for measurement using a UV–Vis spectrophotometer, and the solution that was taken out was replaced with an equal volume of a fresh solution of 3.5 wt% NaCl. The time intervals were 1, 5, 10, 20, and 30 min, followed by testing for every hour until 7 h, then after 24 h and 48 h. The total amount of the Ce^3+^ released was calculated using Eq. (1)^25^.

1 $${F}_{t}={V}_{\!m}{C}_{t}+\mathop{\sum }\limits_{i=0}^{t-1}{V}_{\!a}{C}_{i}$$

Whereas *V*_m_ is the total volume of 3.5 wt% NaCl solutions (100 ml), *C*_*t*_ is the Ce^3+^ concentration in 100 ml at different time intervals (*t*), *V*_a_ is the withdrawal aliquot of 5 ml, and *C*_i_ is the Ce^3+^ concentration in various time intervals (*i*).

The unknown concentration of Ce^3+^ in (1) was measured using a UV–Vis spectrophotometer within the wavelength range of 400–200 nm, whereas the absorbance of Ce^3+^ occurs at a wavelength of 252 nm^26^. The unknown amount of Ce^3+^ was determined by establishing a calibration graph. The calibration graph is plotted by measuring the absorbance of a series of Ce^3+^ standard solutions and from this graph, the equation of the straight line can be used to determine the unknown concentration of Ce^3+^ (Eq. (2))

2 $$y={mX}+C$$

Whereas *m* is the slope from the calibration graph, *X* is the Ce^3+^ standard concentration, *C* is the intercept, and *y* is the absorbance.

### PEO process

PEO coatings were produced in pulsed bipolar mode, using two DC power supplies of GX series from Analog & Digitale Leistungselektronik GmbH, and a pulse unit from Magplus Stromversogungen GmbH. The specimen was connected to an output of the power supply and submerged in a glass container holding 4 l of electrolyte accompanied by a stainless-steel collar and water coil, which together constituted the counter electrode. The water coil kept the temperature of the electrolyte below 30 °C under continuous agitation of the electrolyte. The electrolyte contained (gl^−1^) 20 Na_2_SiO_3_ and 2 KOH (pH 13.0 at 19 °C, conductivity 14.8 mS cm^−1^). An asymmetric square wave signal was used with anodic and cathodic pulse currents of 180 mA cm^−2^ and 234 mA cm^−2^, respectively. PEO process started with a 30 s voltage ramp to reach the desired anodic and cathodic currents. The applied frequency was 1000 Hz, and 15% duty cycle for a treatment duration of 3 min. Afterwards, the sealing post-treatment was applied to Al-PEO specimens through an immersion process. For that, 50 ml of deionised water containing 10 g of the nanoparticles (HNT or zeolite) was sonicated for 20 min to suspend the nanoparticles. Subsequently, the coatings were immersed in the nanoparticles-containing solution for 10 min. Then, the coatings were dried in the oven at 100 °C for 2 h. Hereafter, the PEO coatings sealed with HNT and zeolite denoted as PEO-HNT and PEO-Zeolite, respectively, and the PEO coatings sealed with Ce-HNT and Ce-Zeolite denoted as PEO-Ce-HNT and PEO-Ce-Zeolite, respectively.

### Characterisation of nanocontainer-inhibitor systems

The intercalation of Ce^3+^ within the nanoparticles was confirmed using scanning electron microscopy (SEM) equipped with energy-dispersive X-ray spectroscopy (EDS) (Quanta 250). The surface charge (zeta potential) was measured using a Zetasizer Malvern Panalytical instrument. The process was carried out at 20 °C by dispersing the nanoparticles in deionized water at different pH ranges between 3 and 10. Measurements were performed in triplicate.

### Characterisation of PEO coatings

The coating surface and cross-sectional microstructures were analysed using SEM-EDS (Quanta 250). Thickness measurements were conducted with an eddy current gauge (Elcometer Limited), averaging 10 measurements taken at different locations. Compositional analysis was carried out by X-ray diffraction using a Malvern Panalytical X’Pert MPD (CuKα = 1.54056 Å) operating at a 3° incident angle, with a 2*θ* scan range between 10° and 90°, a 0.05° step size, and a 5 s dwell time. Phase identification was performed using X’pert High Score software and the ICSS PDF4+ database.

### Corrosion evaluation

The corrosion resistance of the produced PEO coatings was evaluated using potentiodynamic polarisation tests and electrochemical impedance spectroscopy (EIS). Both measurements were conducted in a naturally aerated 3.5 wt% NaCl solution at 20 °C. A BioLogic VSP-300 potentiostat and a three-electrode cell system were used for the tests. The coated specimen (11.7 cm^2^) acted as the working electrode, with a silver/silver chloride (Ag/AgCl, 0.210 V vs SHE) reference electrode and a platinum counter electrode. Polarisation measurements were performed after 1 h of exposure, from −300 mV to 1500 mV *vs* OCP at a 0.3 mV s^−1^ scan rate. The current was restricted to a value of 5 mA cm^−2^.

Prior to the EIS measurement, the open circuit potential (OCP) was monitored for 1 h. A 10 mV sinusoidal perturbation vs OCP was applied over a frequency range of 10^5^–10^−2^ Hz after 1, 2, 3, 4, 24, 48, 72, 96 and 120 h immersion in 3.5 wt% NaCl. EIS spectra were analysed and fitted to a model using ZView software (Scribner Associates Inc.), with each element having an error of less than 10% and *χ*^2^ values below 0.003.

## Results and discussion

### Evaluation of nanocontainer-inhibitor systems

**Fig. 1:**
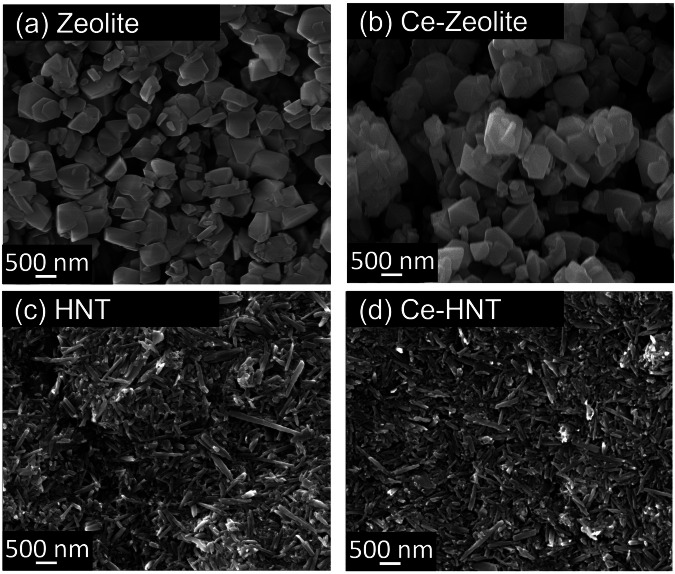
SEM micrographs showing the surface morphology of the evaluated nanoparticles. **a** Zeolite, (**b**) Ce-Zeolite, (**c**) HNT, and (**d**) Ce-HNT.

Figure 1 shows the morphology of zeolite and HNT nanocontainers. Zeolite is composed of hydrated aluminosilicate organised in a 3D cubic structure with particle sizes ranging between 245 and 450 nm. It possesses a negative surface charge due to the substitution of Si^4+^ in its structure by Al^3+^ ions. This charge is balanced by Na^+^ ions located in the porosity, which, depending on their size and geometry, are classified into solidate and super-cages^13,14^. The intercalation of Ce^3+^ within zeolite did not change the structure (Fig. 1b), as its morphology and Si/Al ratio remained constant (Table 1). The intercalation of Ce^3+^ was facilitated by cationic exchange with Na^+^. However, a significant concentration of Na^+^ was still retained in the zeolite, as revealed in the elemental composition analysis (Table 1). The partial exchange is related to the size of the Ce^3+^ (0.23 nm^27^) and/or hydrated Ce^3+^ (0.45 nm^28^), which only fit inside zeolite super-cages (0.74 nm pore opening diameter^29^), while the size of Na^+^ (0.12 nm^27^) is small enough to fit in both super- and solidate cages (0.23 nm pore opening diameter^29^).

**Table 1:** Elemental composition obtained by EDS analysis of zeolite, Ce-Zeolite, HNT, and Ce-HNT

	Composition (at%)					
	Al	Si	O	Na	Ce	Si/Al
Zeolite	7.6	19.2	65.2	7.9	-	2.5
Ce-Zeolite	8.2	20.5	66.5	2.5	2.4	2.5
HNT	15.4	15.1	69.5	-	-	1.0
Ce-HNT	15.6	15.6	68.3	-	0.5	1.0

HNT has a rolled cylindrical structure with a length ranging between 160 and 700 nm (Fig. 1c). The presence of alumina and silica layers, along with water, induces a disordered arrangement, causing them to curve and roll into multilayer tubes^18^. HNT has a hollow interior, and the dimension of its inner lumen can only be observed by TEM, which can range between 10 and 20 nm^19,20^. This allows the accommodation of both Ce^3+^ and hydrated Ce^3+^ ions. HNT possesses two opposite charges; its interior is positively charged due to the aluminium hydroxide group, while its external surface is negatively charged due to the siloxane or silanol groups at 3 < pH < 8.5^21,22,24^. Therefore, when intercalating Ce^3+^, it electrostatically bonds to the external part of HNT remaining adsorbed or immobilised. Similar to zeolite, the intercalation of Ce^3+^ did not induce any changes to the HNT structure (Fig. 1d), and the Si/Al ratio also remained constant, as shown in Table 1.

**Fig. 2:**
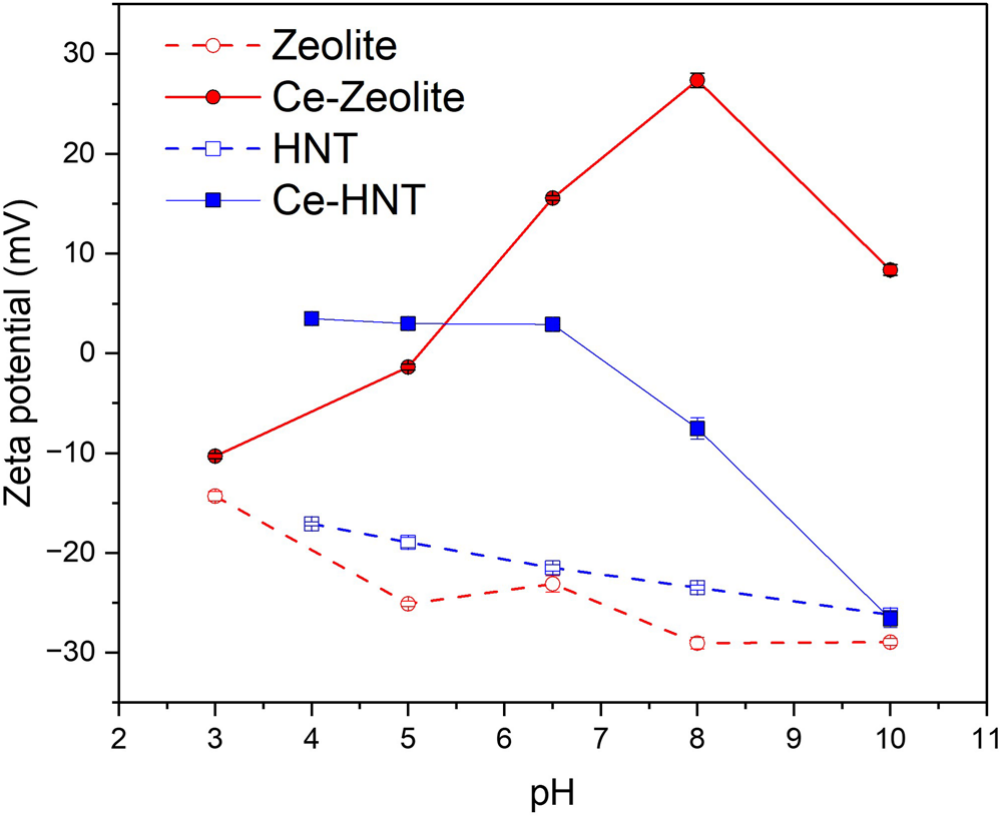
Zeta potential of zeolite, Ce-Zeolite, HNT, and Ce-HNT.

The zeta potential of zeolite and HNT was analysed at various pH to determine the surface charge of the nanoparticles (Fig. 2), which is key to evaluating the inhibitor-nanocontainer interactions and, therefore, demining the release triggers for Ce^3+^ at varying pH. The zeta potential of zeolite over the studied pH range (3–10) remained negative, from −14 to −29 mV. This is due to the ≡SiOH and ≡AlOH_2_ surface groups, which can be deprotonated in an alkaline medium, resulting in a negative surface charge. The encapsulation of Ce^3+^ in zeolite raised the zeta potential from −10 to 27 mV from pH 3 to 8, suggesting that Ce^3+^ ions were also adsorbed on the surface at neutral and slightly alkaline environments. In more alkaline conditions, the zeta potential decreased from 27 to 7 mV from pH 8 to 10, suggesting the partial desorption of Ce^3+^ from the surface. This is likely related to the precipitation of Ce-rich compounds as the pH increases. On the contrary, Yan et al.^30^ reported negative zeta potential values for Ce-Zeolite in alkaline environments, but this can be related to the washing steps typically performed after Ce^3+^ intercalation in zeolite, which effectively eliminates any adsorbed Ce^3+^ from the zeolite surface. In this study, the washing steps were not carried out, suggesting the positive zeta potential reported is due to the adsorption of Ce^3+^ on the surface of zeolite. These adsorbed ions are then released in an alkaline medium, and some of Ce^3+^ would remain in the super-cages within the zeolite structure.

HNT is composed of aluminosilicates with a negative charge in the exterior and a positive charge in its interior^21,22,24^. Within the studied pH range of 4–10, the zeta potential decreased from −17.1 to −26.2 mV, indicating HNT has a negative surface charge due to the deprotonation of the surface silanol group (≡SiO^−^)^22,23^ (Fig. 2). When Ce^3+^ is encapsulated within HNT, measuring the zeta potential in the pH range 4 to 8 becomes challenging due to its charge being close to zero (3.5, 3.0, and 2.9 mV for pH 4, 5, and 6.5, respectively). This suggests that the presence of Ce^3+^ within the HNT exterior counterbalances its charge in that pH range. However, at an alkaline pH, the zeta potential tends to decrease, reaching a minimum value of −26.6 mV at pH 10, which is close to the reported value for hollow HNT. This suggests the desorption of the Ce^3+^ ions from the HNT surface in alkaline conditions.

Therefore, it can be concluded that the release of Ce^3+^ from zeolite is more pronounced in acidic conditions, while for HNT is in alkaline conditions. However, in both cases, if the pH is high enough (pH > 10), Ce^3+^ tends to precipitate in the form of Ce(OH)_3_, Ce(OH)_4_, CeO_2_·*x*H_2_O, or Ce_2_O_3_^4,31,32^.

### Release kinetics of the inhibitor-nanocontainer systems

The Ce^3+^ release evaluation was performed in an aqueous 3.5 wt% NaCl solution at various pH. The release mechanism was plotted based on the model proposed by Aguzzi et al.^33^. This model proposed the release of encapsulated species associated with two mechanisms: a fast release from the nanocontainer’s outer surface (desorption) and a slow release from the nanocontainer’s interior (diffusion). The overall release was calculated using Eq. (3)^33^.

3 $${\rm{F}}=\frac{{\rm{C}}_{{\rm{e}}(1)}}{{\rm{C}}_{\infty }}\left[1-\exp \left(\frac{{\rm{-k}}_{{\rm{d}}\left(1\right)}.t}{{\rm{C}}_{{\rm{e}}\left(1\right)}}\right)\right]+\frac{{C}_{{\rm{e}}(2)}}{{C}_{\infty }}\left[1-\exp \left(\frac{{\rm{-k}}_{{\rm{d}}\left(2\right)}.t}{{\rm{C}}_{{\rm{e}}\left(2\right)}}\right)\right]$$

Whereas *C*_e(1)_ and *C*_e(2)_ represent the concentration of Ce^3+^ at equilibrium, k_d(1)_ and k_d(2)_ represent the desorption rate of Ce^3+^ from the outer surface and from the interior of the nanocontainers, respectively. C_∞_ is the total of *C*_e(1)_ and C_e(2)_.

**Fig. 3:**
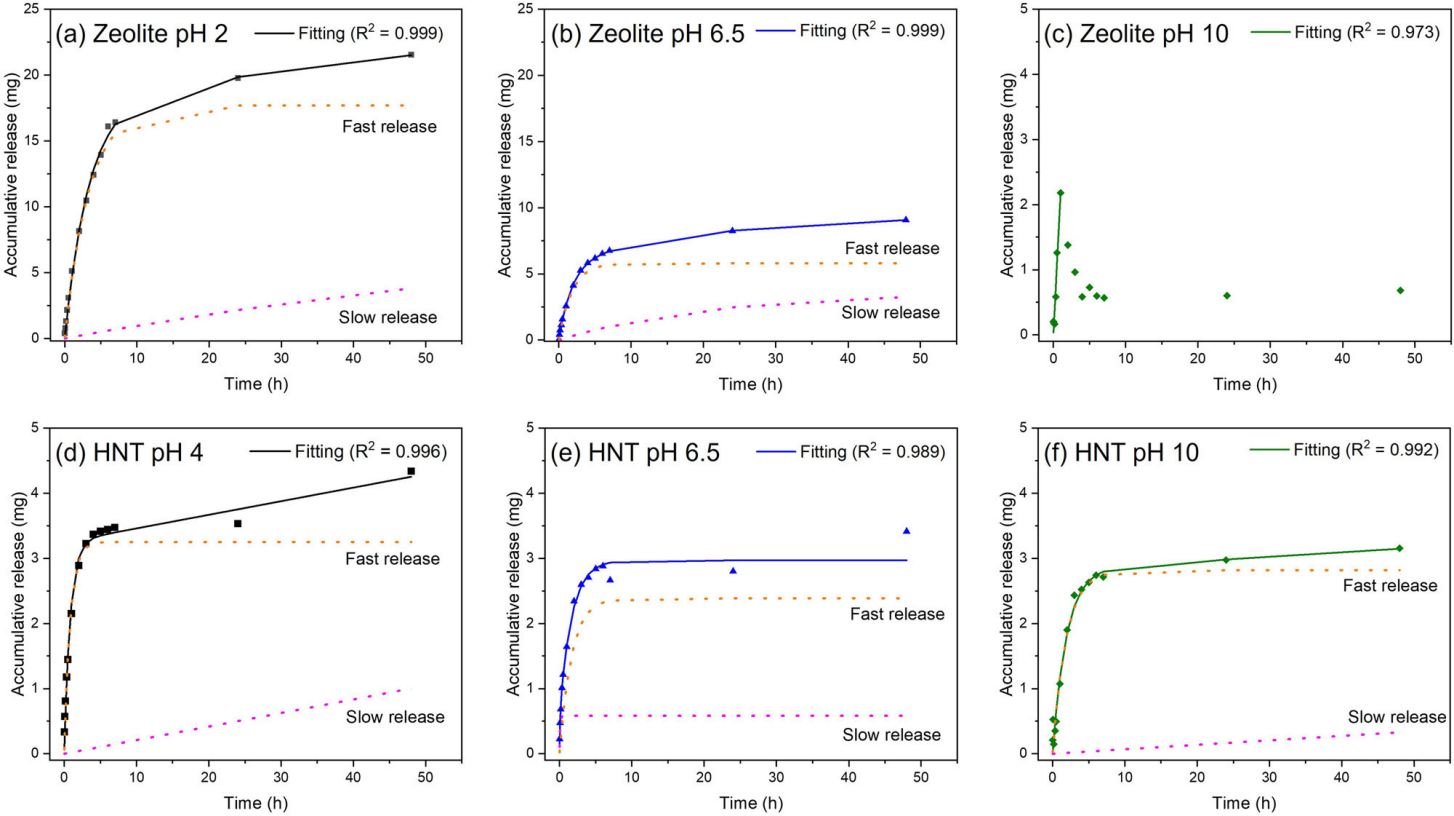
Accumulated Ce³⁺ Release from Zeolite and HNT at Different pH Conditions. **a**–**c** Ce³⁺ release from zeolite and (**d**–**f**) HNT, fitted using the Aguzzi et al. ^33^ model. Dashed lines represent ‘slow’ and ‘fast’ release phases, while the continuous line indicates total release.

Figure 3a–c shows the release profile of Ce^3+^ from zeolite at varying pH 2, 6.5, and 10 in 3.5 wt% NaCl solution. The solid line represents the overall release profile, while the dotted line corresponds to the different release mechanisms. The release of Ce^3+^ at a neutral pH is governed by the cationic exchange with Na^+^ ions. Initially, there is a progressive release of Ce^3+^ until 2 h, followed by a stable release of Ce^3+^ until 48 h (Fig. 3b). The initial fast release was attributed to the desorption of Ce^3+^ adsorbed on the surface of zeolite, and the slow-release duo to the diffusion from zeolite super-cages. At acidic pH, the release of Ce^3+^ was intensified due to the higher concentration of protons (H^+^) that aid in the cationic exchange^26^. These results agree with the zeta potential measurements, which were carried out in deionised water without NaCl (Fig. 2)^34,35^. At an alkaline pH, the surface groups in zeolite deprotonate, resulting in a negative surface charge that promotes the interaction with Ce^3+^, therefore these conditions are not favourable for the release of the inhibitor. Consequently, it was not possible to fit the release data using the Aguzzi et al. model^33^ for testing times above 1 h. The partial release within 1 h was attributed to the desorption of Ce^3+^, and afterwards, no release was observed. The release of Ce^3+^ from zeolite is strongly related to the concentration of the tested solution, as reported by Zadeh et al. ^26^ They observed that at a neutral pH, the release progressed rapidly in concentrated NaCl solution (21.7 wt%) compared to the dilute solution (2.17 wt%), while the solution pH controls the rate of the cationic exchange. This is in agreement with the presented results, where the release kinetics is much faster at acidic pH compared to neutral and alkaline pH.

Figure 3d–f shows the release profile of Ce^3+^ from HNT at varying pH 4, 6.5, and 10 in 3.5 wt% NaCl solution. The slightly higher release profile at pH 4, is attributed to the presence of the protons, which aid the liberation of Ce^3+^. However, at neutral pH and alkaline environments, the release is mainly associated with Na^+^ ion exchange processes. This suggests that in the presence of NaCl, the release mechanism in HNT mainly depends on the concentration of ions in solution. It is worth noting that in the absence of NaCl, changes in pH would govern the release as suggested from the zeta potential analysis. Similar results were reported by Xu et al.^36^, who observed that the release of benzotriazole inhibitor was similar for pH 7 and 10 and slightly faster at pH 4. Pasbakhsh et al.^37^ observed that HNT has a negative zeta potential when tested in a concentrated KCl solution (0.01 M) for a pH range between 4 and 12. This confirms the influence of salts on the inhibitor release behaviour from HNT. It is worth noting that fitting the release profile for Ce-HNT showed similar results considering having two release steps (desorption and diffusion) or one release step. Thus, confirming Ce^3+^ is present in the external part of HNT.

### PEO cell voltage vs. treatment time response

**Fig. 4:**
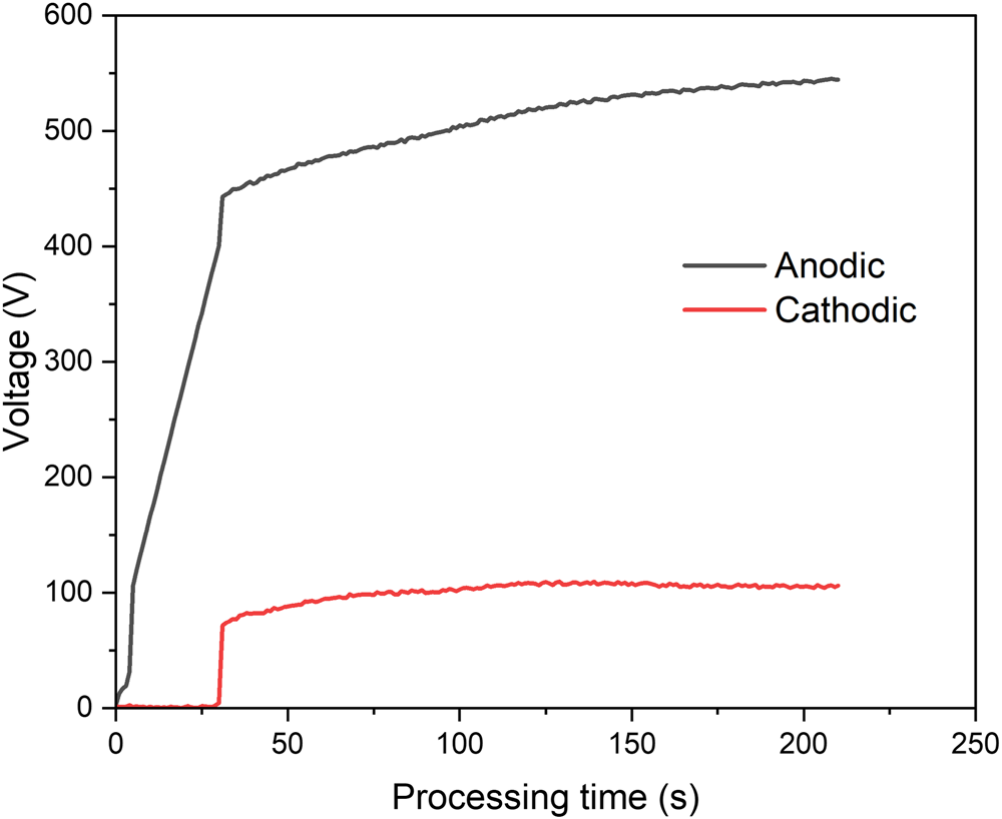
Anodic and cathodic voltages vs processing time for PEO treatment. PEO coatings morphology and composition.

Figure 4 shows the voltage response with time for the PEO process conducted on A1050 aluminium alloy. The first 30 s of the process was voltage ramping up to 400 V, and subsequently the process progressed in current control mode. Three stages were identified during the PEO process. In the first stage (I), a rapid surge in the voltage was observed that is linked to the formation of a passive layer on the substrate, which was also accompanied by the formation of gas bubbles. The second stage (II) commences at 443 V, wherein the anodic and cathodic voltages raised slowly, and orange microdischarges were observed covering the substrate surface. The third stage (III) starts at 522 V, and the rate of voltage rise was further reduced, and the number of microdischarges decreased, but their size was enlarged and lasted for a longer duration, which indicates the increase in the coating thickness^11^.

**Fig. 5:**
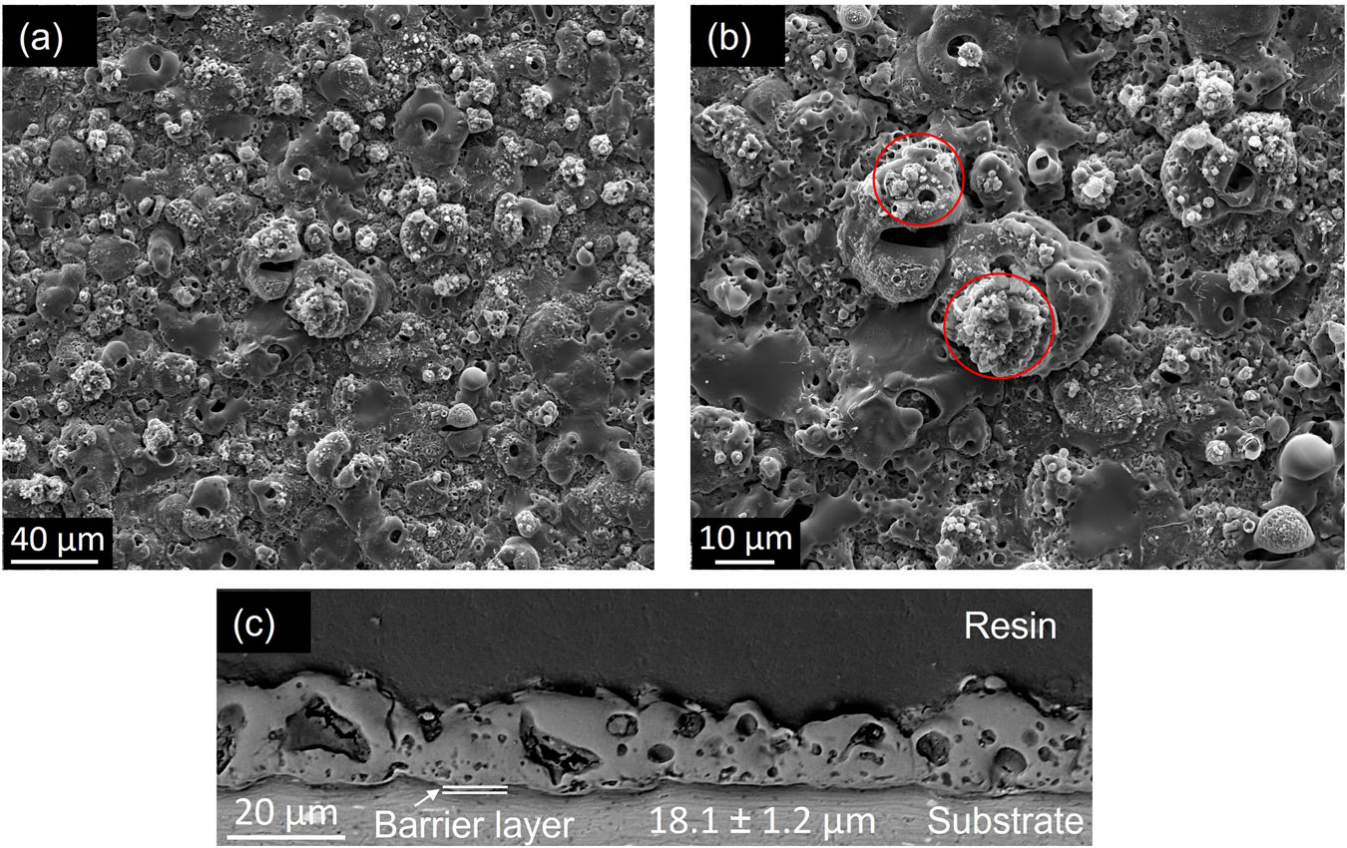
SEM micrographs of the PEO coating. **a**, **b** Surface morphology at different magnifications, and (**c**) cross-sectional micrograph.

Figure 5 illustrates the surface and cross-sectional morphology of the un-sealed PEO coatings. The surface is characterised by open porosity surrounding the discharge channels. These discharge channels are produced by the deposition of the expelled molten oxides, which are then solidified by the cooling electrolyte during the PEO process^38^. The cauliflower-like feature and the nodular structure surrounding the discharge channels are produced from the reaction of the silicon content of the electrolyte with the molten oxide (shown in circle insets in Fig. 5b). The coating consists of two main layers: an outer layer with variable porosity and a thin compact barrier layer that adheres to the substrate. The existence of the porous oxide layer is associated with the liberation of the trapped oxygen and hydrogen gas from the molten alumina as it gets solidified^39^.

**Fig. 6:**
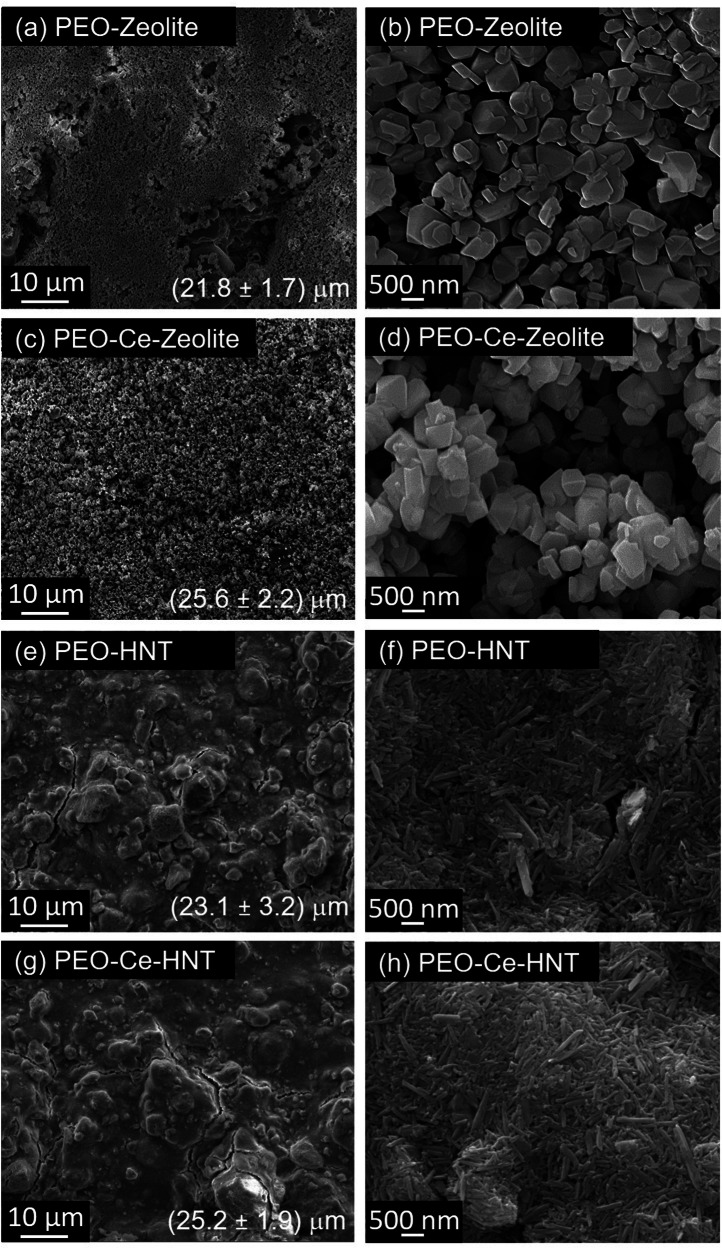
SEM micrographs of the surface morphology of sealed PEO coatings shown at different magnifications. **a**, **b** PEO- Zeolite, (**c**, **d**) PEO-Ce-Zeolite, (**e**, **f**) PEO- HNT, and (**g**, **h**) PEO-Ce-HNT.

The surface morphology of post-treated PEO coatings is shown in Fig. 6. It is clear from the SEM micrographs that the nanocontainers cover the surface of the coatings by forming a top layer with a different level of coverage as it can be deduced from the increase in the coating thickness values (Fig. 6). In PEO-Zeolite, the coverage is almost complete, however, some unsealed areas can still be identified, while in PEO-HNT, the sealing is complete across the surface, although some cracks are evident, possibly formed during the drying process. Some agglomerations are also evident. The HNT sealing appears to be more compact, while in zeolite some spaces can be identified between clusters of particles. This is possibly related to the combination of the larger size of 3D structured zeolite nanoparticles, compared to the 2D and smaller HNT nanoparticles, which allows a more compact arrangement. Coatings post-treated with encapsulated Ce^3+^ showed some morphological differences in PEO-Ce-Zeolite (Fig. 6c), which resulted in a higher level of coverage, and the Ce-Zeolite nanoparticles had a higher degree of agglomeration. Possibly, the presence of Ce^3+^ adsorbed on the zeolite’s outer surface affected the charge distribution, causing zeolite to pack and aggregate non-homogeneously on the coating surface. The coating morphology of Ce-HNT resembles that of HNT, suggesting the presence of Ce^3+^ did not alter the surface morphology of the coating.

Table 2 gathers the elemental compositional analysis of the developed coatings. Unsealed PEO is mainly formed by Al and Si in the form of oxides, with low concentrations of Na and K originated from the electrolyte. Zeolite-containing coatings showed a lower concentration of Al coming from the PEO coating, due to the masking effect of the sealing layer; while the concentration of Si remained in the same range, which is related to the Si present in zeolite, which also correlates with the increase in Na concentration. In HNT-containing coatings, an increase in Al was observed related to the presence of Al within the HNT. In both PEO-Ce-Zeolite and PEO-Ce-HNT, Ce was detected, although the concentration was higher in PEO-Ce-Zeolite, which is associated with zeolite’s ability to host Ce^3+^ both on the surface and inside the porosity.

**Table 2:** Elemental composition obtained by EDS analysis of PEO coatings with zeolite and HNT with encapsulated Ce^3+^

	Composition (at%)					
	Al	Si	O	Na	K	Ce
PEO	12.8	20.9	64.2	1.4	0.7	
PEO-Zeolite	8.5	21.8	62.7	6.8	0.3	
PEO-Ce-Zeolite	8.0	20.9	66.2	2.9	0.1	2.0
PEO-HNT	15.5	14.8	69.4	0.2	0.1	
PEO-Ce-HNT	14.5	13.5	71.8	0.1	-	0.1

**Fig. 7:**
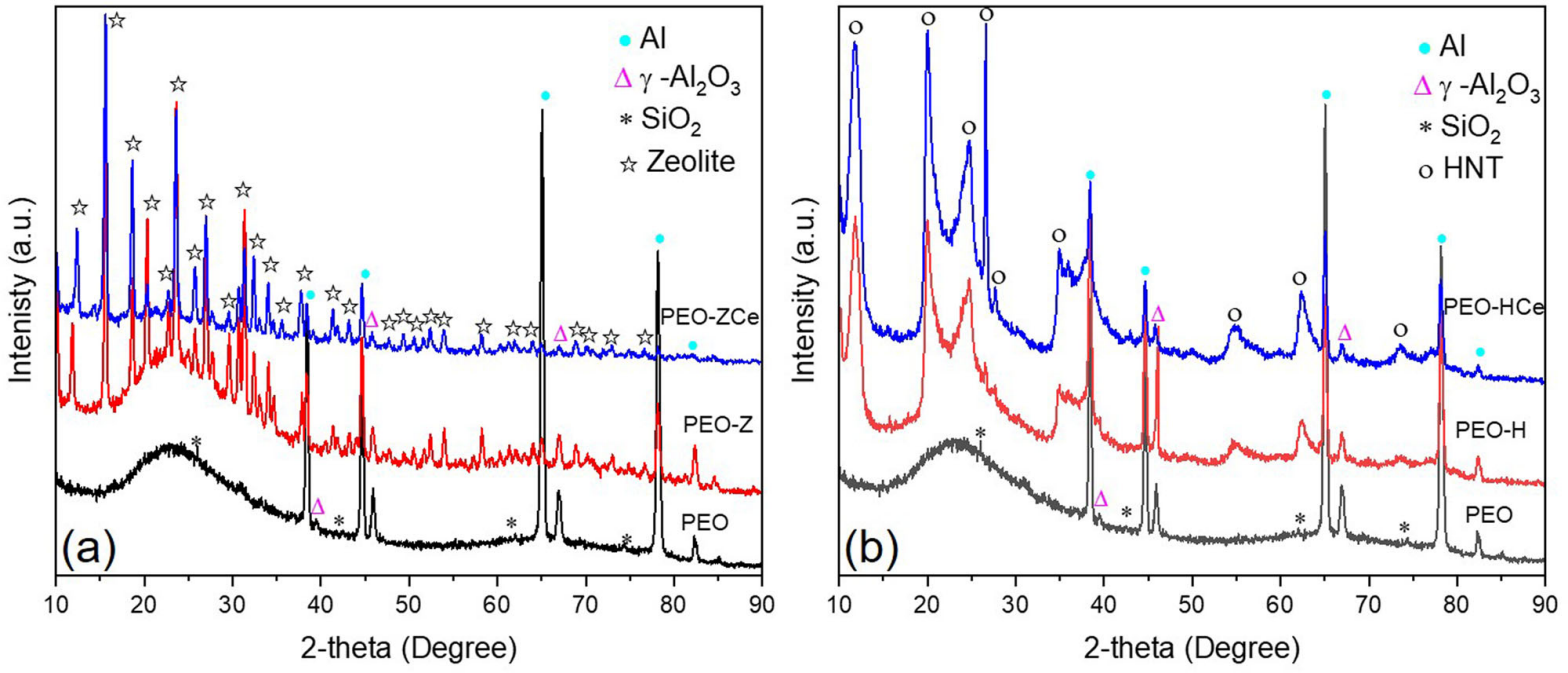
XRD patterns of sealed and unsealed PEO coatings. **a** PEO coating with Zeolite, and (**b**) PEO coating with HNT.

PEO coatings are mainly composed of γ-Al_2_O_3_ and SiO_2_, as revealed from XRD analysis (Fig. 7), which is in accordance with the EDS analysis (Table 2). The predominant formation of γ-Al_2_O_3_ over α-Al_2_O_3_ phase is related to the short PEO processing time and high frequency, which reduce the plasma temperature and therefore inhibit the transition from γ-Al_2_O_3_ to α-Al_2_O_3_^40^. An amorphous SiO_2_ phase was also observed between 20° and 35° 2-theta which is commonly formed in concentrated silicate-based electrolyte^41^. The diffraction patterns of the PEO coatings after being subjected to the posttreatment showed the characteristic peaks of the nanoparticles; the intensity of the PEO coating forming phases decreased, due to the masking effect of the sealing layer.

### Corrosion evaluation: potentiodynamic polarisation measurements

**Fig. 8:**
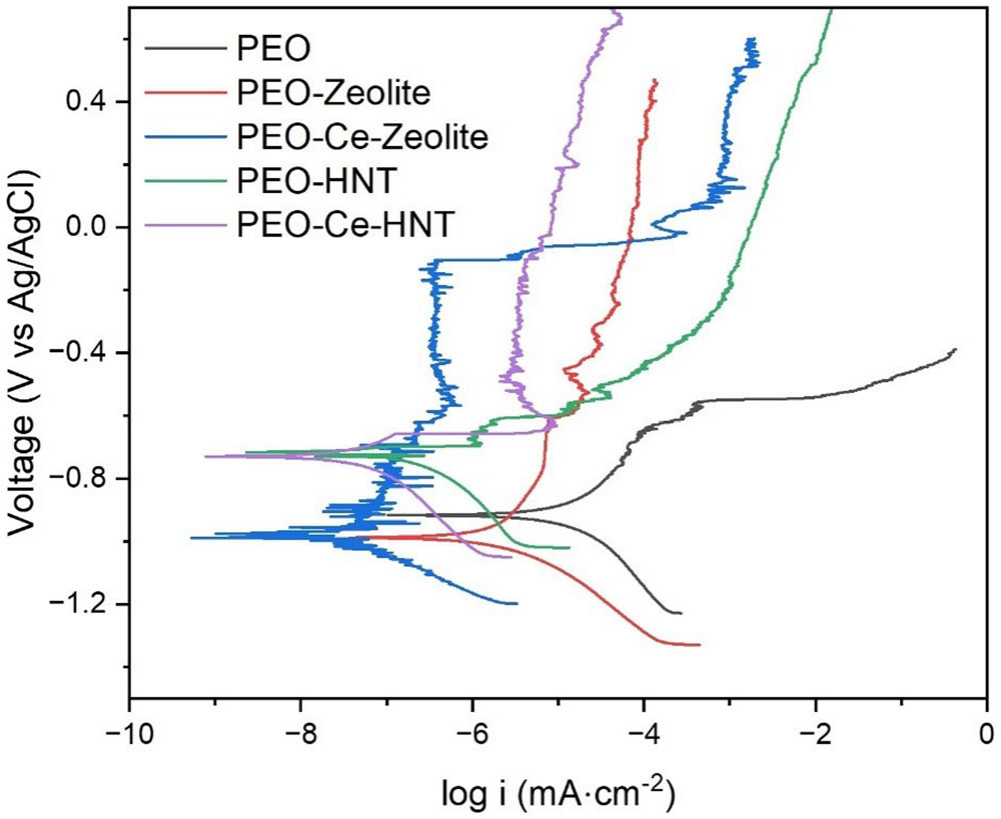
Potentiodynamic polarisation curves of PEO coatings sealed with zeolite, Ce-Zeolite, HNT, and Ce-HNT after 1 h of immersion in naturally aerated 3.5 wt% NaCl solution.

Electrochemical techniques were used to assess the corrosion resistance of the produced coatings in naturally aerated 3.5 wt% NaCl solution. Figure 8 shows the potentiodynamic polarisation response of the coatings after 1 h of immersion. In all cases, the corrosion performance of the coatings sealed with the nanoparticles was higher than the unsealed coating, as deduced from the lower icorr values. PEO-HNT achieved a two-order of magnitude reduction in icorr compared to the particle-free coating, while PEO-Zeolite showed an improvement of one order of magnitude. The enhanced reduction in icorr for PEO-HNT can be associated with the slightly higher coating thickness (21.8 ± 1.7 and 23.1 ± 3.2 μm, for PEO-Zeolite and PEO-HNT, respectively, Fig. 6), a higher level of nanoparticle compactness within the sealing layer and the ability of HNT to trap corrosive ions (Cl^−^) that can easily migrate through the coating porosity, damaging the barrier layer and promoting corrosion initiation. As described in section 0, the inner layer of HNT is positively charged, while the outer layer is negatively charged (3 < pH < 8.5) which allows the electrostatic encapsulation of negatively charged species.

To evaluate the nanoparticles’ capacity to interact with Cl^-^ ions, EDS analysis was performed on zeolite and HNT nanoparticles after immersion in a naturally aerated 3.5 wt% NaCl solution for 1 and 96 h. The EDS analysis presented in Table 3 clearly shows the presence of Cl^−^ after exposure to the NaCl solution, which is the sole source of Cl in the system. It confirms the preferential encapsulation of Cl^-^ in HNT, with 1.53 and 2.15 wt% Cl identified for zeolite and HNT, respectively. The encapsulation of negatively charged species within the inner layers of HNTs has been extensively documented in the literature^21,22,24,42^. While the present work indicates an increase in Cl content, the exact mechanism of entrapment remains inferred based on established behaviours of HNTs. It is worth noting that the concentration of Na also increases, which is in agreement with the ion exchange release mechanism discussed in the Release Kinetics section.

**Table 3:** EDS analysis of HNT and zeolite nanoparticles after 1 and 96 h of immersion in 3.5 wt% NaCl

Composition (wt%)							
Nanocontainer	time (h)	C	O	Al	Si	Na	Cl
HNT	1	36.3	39.43	12.00	12.02	-	0.22
	96	24.72	45.40	13.15	13.01	1.58	2.15
Zeolite	1	8.99	48.09	9.46	25.43	7.89	0.15
	96	20.80	43.72	7.23	19.58	7.15	1.53

The i_corr_ values of PEO coatings containing encapsulated inhibitors were reduced further, and in both cases, the values were in a similar range. These values are within the same order of magnitude as those reported for other PEO coatings formed on Al and sealed with corrosion inhibitors (MBT, 8-HQ, and polyacrylic acid)^43^. The improvement is related to cerium inhibition activity, leading to the formation of cerium oxides/hydroxides, which enhance the passive properties of the coating and prevent further degradation. PEO-Ce-HNT also shows some signs of breakdown and repassivation that are likely related to the cerium inhibition effect.

It is worth noting that the amount of released Ce^3+^ in zeolites (21.51 mg in 48 h) was considerably higher than in HNT (4.33 mg in 48 h); however, both show a similar improvement in corrosion resistance, suggesting that small amounts of inhibitor are sufficient to significantly impact the overall corrosion properties.

### Corrosion evaluation: electrochemical impedance spectroscopy (EIS)

**Fig. 9:**
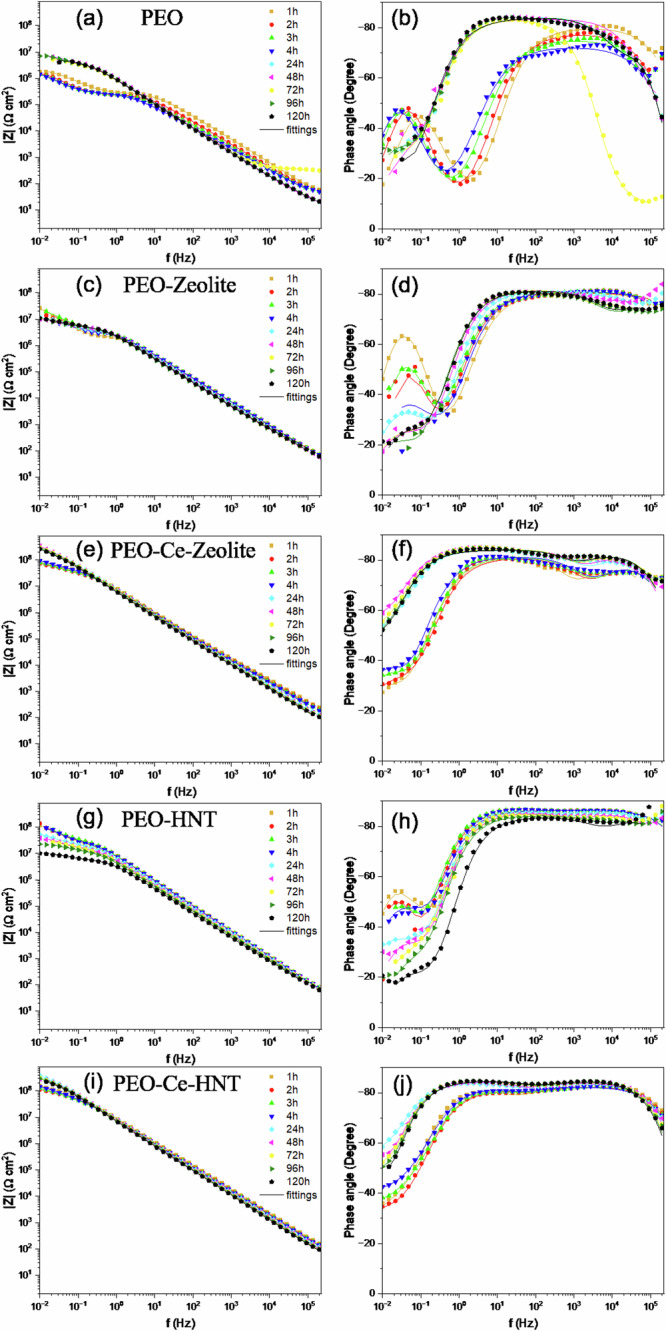
EIS Bode plots of PEO coatings after immersion in naturally aerated 3.5 wt% NaCl solution for up to 120 h. **a**, **b** PEO, (**c**, **d**) PEO-Zeolite, (**e**, **f**) PEO-Ce-Zeolite, (**g**, **h**) PEO-HNT, and (**i**, **j**) PEO-Ce-HNT.

**Fig. 10:**
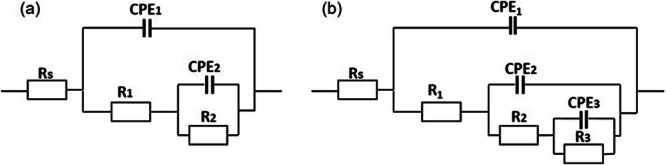
Equivalent circuits used to fit EIS data for PEO coatings at different immersion times. **a** PEO, PEO-Zeolite, and PEO-HNT up to 4 h of immersion, and (**b**) PEO, PEO-Zeolite, and PEO-HNT after 24–120 h of immersion; PEO-Ce-Zeolite and PEO-Ce-HNT were also fitted using circuit (**b**) for 1–120 h of immersion.

The corrosion evaluation of PEO coatings containing zeolite or HNT was evaluated using electrochemical impedance spectroscopy in a naturally aerated 3.5 wt% NaCl solution for 120 h (Fig. 9). Figure 10 illustrates the equivalent circuits used to fit the EIS spectra, and Supplementary Tables 1–5 summarise the electrochemical measurements extracted from the fittings. The symbol Rs denotes the resistance of the electrolyte; Constant Phase Elements (CPEs) were used instead of ideal capacitors to account for the inhomogeneity of the coatings. Two equivalent circuits were used to fit the corrosion behaviour of PEO, PEO-Zeolite, and PEO-HNT coatings as a function of the immersion time. For short immersion times (1–4 h), two-time constants were considered, related to the capacitive and resistive behaviour of the outer and the inner barrier layer CPE_1_/R_1_ and CPE_2_/R_2_, respectively. For immersion periods lasting between 24 and 120 h, three-time constants were employed to address the corrosion activity at the interface between the metal coating, associated with the capacitive behaviour of the double layer and the charge transfer resistance (CPE_3_/R_3_). Coatings containing encapsulated Ce^3+^ (PEO-Ce-Zeolite and PEO-Ce-HNT) were fitted using three-time constants. At short immersion times (<4 h), these corresponded to the capacitive/resistive behaviour of the outer and barrier layer and the mass transport phenomena associated with Ce^3+^ diffusion from the outer layer of the coating towards the corrosion active sites. At longer immersion times, the third time constant at low frequencies was associated with the precipitation of a protective cerium-rich corrosion product layer (CPE_3_/R_3_). The produced cerium compounds can be represented as a capacitive and resistance behaviour similar to cerium-based conversion coatings^44^.

**Fig. 11:**
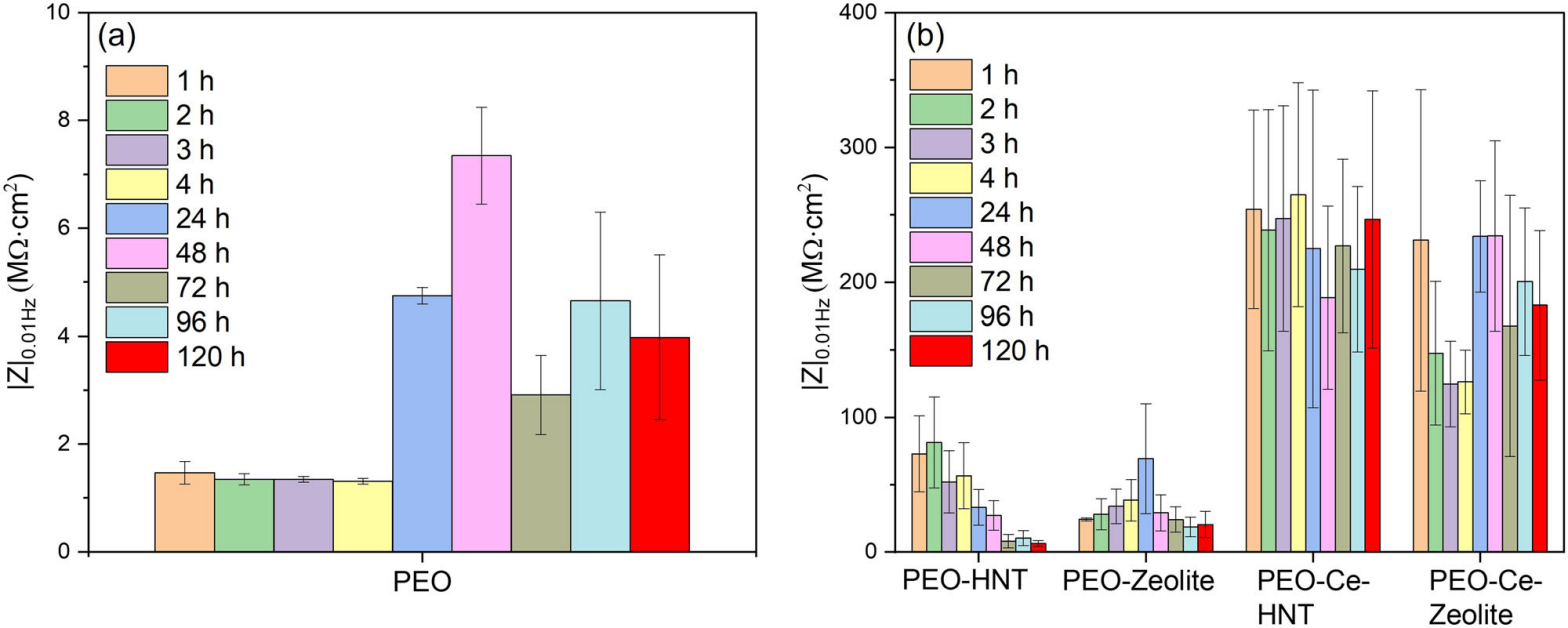
Impedance modulus of PEO coatings at 0.01 Hz as a function of immersion time in 3.5 wt% NaCl solution. **a** PEO coating and (**b**) sealed PEO coatings with nanoparticles.

The impedance of the unsealed PEO coating (Fig. 9a) remained relatively constant up to 4 h of immersion as observed in the |Z|_0.01 Hz_ plot (Fig. 11a) and as deduced from the constant diameter of the capacitive loop in the Nyquist plot (Supplementary Fig. 2). R_1_ (Supplementary Table 1), also remained relatively high. After 24 h, a sharp decrease in R1 was observed, attributed to the penetration of the electrolyte within the coating open porosity and, ultimately, reaching the substrate. This leads to the deterioration of the barrier layer and the onset of corrosion. Interestingly, the total impedance of the system (Fig. 9a) started to increase slightly over time, as deduced from the larger diameter of the capacitive loop in the Nyquist plot (Supplementary Fig. 2), due to the formation of a partially protective corrosion product layer. Some fluctuations were observed, which are related to the cyclic precipitation and dissolution of these products^11,45^.

PEO coatings containing hollow nanoparticles (PEO-Zeolite and PEO-HNT) (Fig. 9c, d and g, h), showed a higher corrosion resistance both at short and long immersion times, which is attributed to the sealing effect provided by the presence of nanoparticles on the surface of the coatings, which tends to increase in R1 (Supplementary Tables 2, 3). This is also evident from the larger diameter of the capacitive loops in the Nyquist plot and the higher |*Z*|0.01 Hz values (Supplementary Fig. 2b, d, and Fig. 11b, respectively). Nevertheless, similarly to the unsealed PEO, within 24 h, the electrolyte penetrated the coatings, leading to the initiation of corrosion and the formation of partially protective corrosion products, although the overall |*Z*|0.01 Hz remained considerably higher compared to the particle-free coatings, which evidences the benefits of the sealing post-treatment. PEO-HNT exhibited greater corrosion resistance compared to PEO-Zeolite, which might be related, amongst other factors, to the ability of HNT to retain corrosive electrolyte ions (Cl^−^), which are known to promote the hydrolysis of Al^3+^ in the proximity of the anodic sites. Cl^−^ ions disrupt the protective oxide layer on aluminium and form soluble aluminium-chloride complexes, enhancing the hydrolysis process. This interaction not only prevents the precipitation of aluminium hydroxides but also increases the concentration of H^+^ ions, leading to localised acidification. The acidic environment further accelerates aluminium dissolution and hydrolysis, creating a feedback loop that promotes the corrosion process^46^.

Coatings containing encapsulated Ce^3+^ exhibited significantly superior corrosion performance (Fig. 9e, f, i, j). The |*Z*| at 0.01 Hz decreased progressively during the first 4 h of immersion; however, after 24 h, the corrosion resistance increased considerably, recovering to the values observed at the start of the test. In the initial stage (1–4 h), Ce^3+^ ions diffuse from the outer layer of the coating to active corrosion sites. This mass transport phenomenon was identified at low frequencies. Typically, such diffusion-related processes are modelled by a Warburg element in equivalent circuits, which represents semi-infinite diffusion. However, when the diffusion process is not ideal or is restricted (e.g., finite diffusion through a coating), it can be approximated by a Constant Phase Element (CPE) with an n value near 0.5, which reflects the non-ideal capacitive behaviour associated with diffusion (CPE_3_/R_3_).

In the case of PEO-Ce-HNT, *n* ranged from 0.5 to 0.57 (Supplementary Table 5), indicating diffusion-controlled behaviour similar to a finite Warburg process. In contrast, for PEO-Ce-Zeolite, *n* values were slightly higher, reaching values in the range 0.67–0.87 during the first 1–4 h of immersion, suggesting a more capacitive behaviour with less pronounced diffusion limitations. This indicates that mass transport restrictions were more evident in PEO-Ce-HNT, which could be attributed to the faster release kinetics of Ce^3+^ from zeolite compared to HNT (Fig. 3), which increases the concentration gradient and thus promotes the diffusion process more efficiently. This faster release of Ce^3+^ from zeolite may also be promoted by the local acidification triggered by hydrolysis reactions in Cl^−^-rich environments. After 24 h, the corrosion resistance increased considerably, returning to the values observed at the beginning of the test (Fig. 11b). Once the Ce^3+^ ions reached the metal interface, they precipitated on the active cathodic sites, hindering further reduction of oxygen and therefore inhibiting corrosion propagation, and at the same time offering further protection against corrosion.

**Fig. 12:**
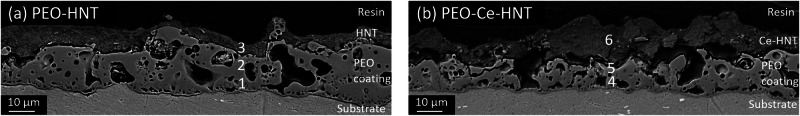
SEM cross-sectional micrographs of PEO coatings after 120 h EIS test in naturally aerated 3.5 wt% NaCl solution. **a** PEO-HNT and (**b**) PEO-Ce-HNT.

To study the corrosion failure mechanism, cross-sectional characterisation of the developed coatings was performed after a 120-h EIS corrosion test in 3.5 wt% NaCl solution (Supplementary Fig. 4). The presented multilayer coating system corrodes at the interface between the coating and substrate, and this interfacial corrosion is not visible from the surface morphology, making it challenging to locate the corrosion front for cross-sectional analysis. The plan view of PEO-HNT-Ce reveals a relatively dense surface. Notably, the cracks initially observed in the HNT-containing coating (Fig. 6) disappeared after the immersion test, and no new cracks were identified. This absence of cracks may be associated with the formation of hydrated corrosion products at cracks and fissures, providing further semi-protective properties. In contrast, the PEO-Zeolite-Ce coating shows a less dense film with multiple access points for the electrolyte. The presented cross-sectional micrographs (Fig. 12) represent two randomly selected locations of the coating and are, therefore, not representative of the most damaged areas of the coating. Nevertheless, they provide some relevant information. After the corrosion tests, the sealing layer remained relatively well adhered to the coating (Fig. 12a). However, in some areas near the active corrosion sites, delamination was observed (Fig. 12b). EDS analysis (Table 4) further confirmed the presence of Cl in the HNT-containing layer.

**Table 4:** EDS analysis at different locations (points shown in Fig. 12) of PEO-HNT and PEO-Ce-HNT coatings

	Composition (at%)						
	O	Al	Si	Na	K	Ce	Cl
Point 1	56.0	34.4	8.8	0.4	0.4	-	0
Point 2	61.5	20.3	17.6	0.2	0.4	-	0
Point 3	59.9	19.0	20.5	0.2	0	-	0.4
Point 4	52.2	38.5	8.5	0.4	0.2	0	0.2
Point 5	61.6	13.8	24.0	0.3	0.3	0	0
Point 6	55.9	20.3	22.5	0.2	0	0.1	1.0

In both systems, the local decrease in impedance is associated with the failure of the sealing layer, which HNT seems to delay to a greater extent, slowing down the permeability of the electrolyte through the coating. Subsequent increases in corrosion resistance are associated with the inhibition effect of the Ce^3+^ released from the nanocontainers, which diffuse towards the active corrosion sites, precipitating at the cathodic site in the form of cerium oxides/hydroxides, which enhance the passive properties of the coating and prevent further degradation^47^. Despite the mass transport limitation of Ce^3+^ in PEO-Ce-HNT, this system showed a slightly better corrosion performance than PEO-Ce-Zeolite, which could be related to the combination effect of a denser layer (Fig. 6 and Supplementary Fig. 4) and HNT’s ability to trap Cl^−^ ions through electrostatic interactions, thereby mitigating their detrimental effects on the aluminium substrate by reducing chloride-induced pitting and corrosion propagation. Consequently, this trapping mechanism enhances the coating’s protective capabilities, even with the slower release of inhibitors.

## Supplementary information

Supplementary material

## Data Availability

Data sets generated during the current study are available from the corresponding author on reasonable request.
