# Supplementary material for: Multilayer PEO coatings with encapsulated cerium for active corrosion protection of aluminium
Source: Npj Mater Degrad. 2025 Mar 7;9(1):24. doi: 10.1038/s41529-025-00560-3 (PMC11888989; doi:10.1038/s41529-025-00560-3)
Supplement: Supplementary file 1 — Supplementary material [file 41529_2025_560_MOESM1_ESM.pdf]

Supplementary material

Multilayer PEO coatings with encapsulated cerium for active corrosion protection of aluminium

Safiya Al Abri[1,2], Tess Knowles[1,2], Yitao Pan[1,2], Aleksey Yerokhin[1,2], Beatriz Mingo[1,2]*

[1]*Department of Materials, The University of Manchester, Oxford Road, M13 9PL Manchester, United Kingdom*
[2]*Henry Royce Institute, Oxford Road, M13 9PL Manchester, United Kingdom*
**beatriz.mingo@manchester.ac.uk*

1. XRD Characterisation of nanocontainer-inhibitor systems

The XRD diffraction patterns of zeolite and HNT are displayed in Supplementary Figure 1. Zeolite nanocontainers had distinct peaks corresponding to their structure at 6.3, 10.2, 15.7, 23.7, 27.1, and 21.4° 2-theta[1-4]. Similar diffraction patterns were also observed for Ce-zeolite; the peak position remains unchanged, suggesting the intercalation of Ce^{3+} did not induce any significant changes in the zeolite structure. Two additional peaks (insert in Supplementary Figure 1a) were detected at 14.4°, corresponding to the incorporation of larger atoms within the zeolite structure, which in this study corresponds to the intercalation of Ce^{3+} and a peak at 12.4° attributed to the insertion of water molecules[5]. The addition of Ce^{3+} also resulted in a slight reduction in the peak intensity, ascribed to a minor reduction in zeolite crystallinity[6-8].

The XRD diffraction pattern of HNT (Supplementary Figure 1b) exhibited peaks at 11.6 °, 19.9 °, 24.8 °, 26.6 °, 34.9 °, 54.8 °, and 62.4° 2-theta[3]. The peak detected at an angle of 11.6° corresponds to a basal plane of 001, that is equivalent to a d-value of 7.2 Å, which is attributed to a multilayer tubular morphology composed of aluminosilicate [9]. The existence of peaks at 19.9° and 24.8° 2-theta indicates that HNT is in the dehydrated state, with an n value in the chemical formula ($Al_2Si_2O_5(OH)_4{\cdot}nH_2O$) equal zero[10]. The peak at 26.6° 2θ suggests the presence of SiO_2[3,11-13]. The diffraction pattern of Ce-HNT revealed similar patterns to HNT, indicating that the intercalation of Ce^{3+} did not modify the crystal structure of HNT. The intercalation of Ce^{3+} within HNT did not show any additional peaks corresponding to Ce^{3+} as observed in zeolite. Nevertheless, an insignificant reduction in the intensity of the peaks was detected when Ce^{3+} has been introduced. This suggests that Ce^{3+} might have been incorporated in the external region of HNT[14].

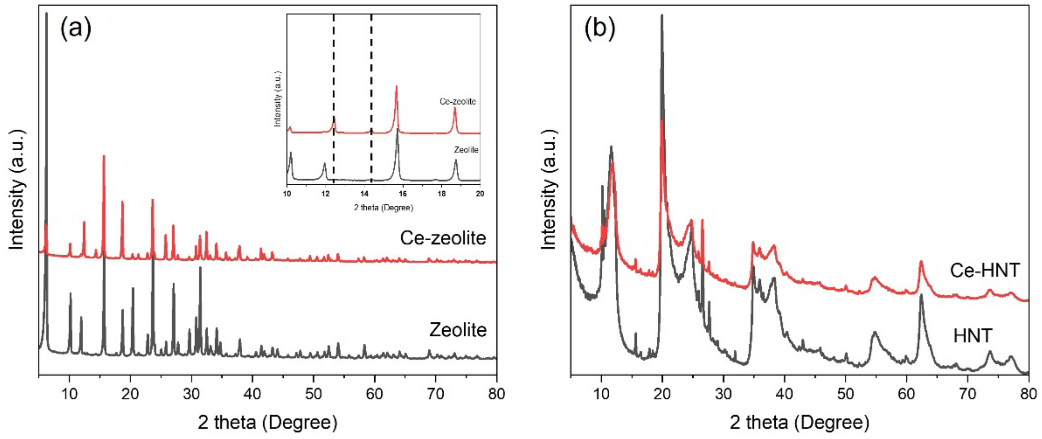

Supplementary Figure 1. XRD patterns of Zeolite and Ce-Zeolite (adapted from[4] licensed under CC BY 4.0) (a), and HNT and Ce-HNT (b).

2. Corrosion evaluation

2.1 Electrochemical impedance spectroscopy Nyquist plots

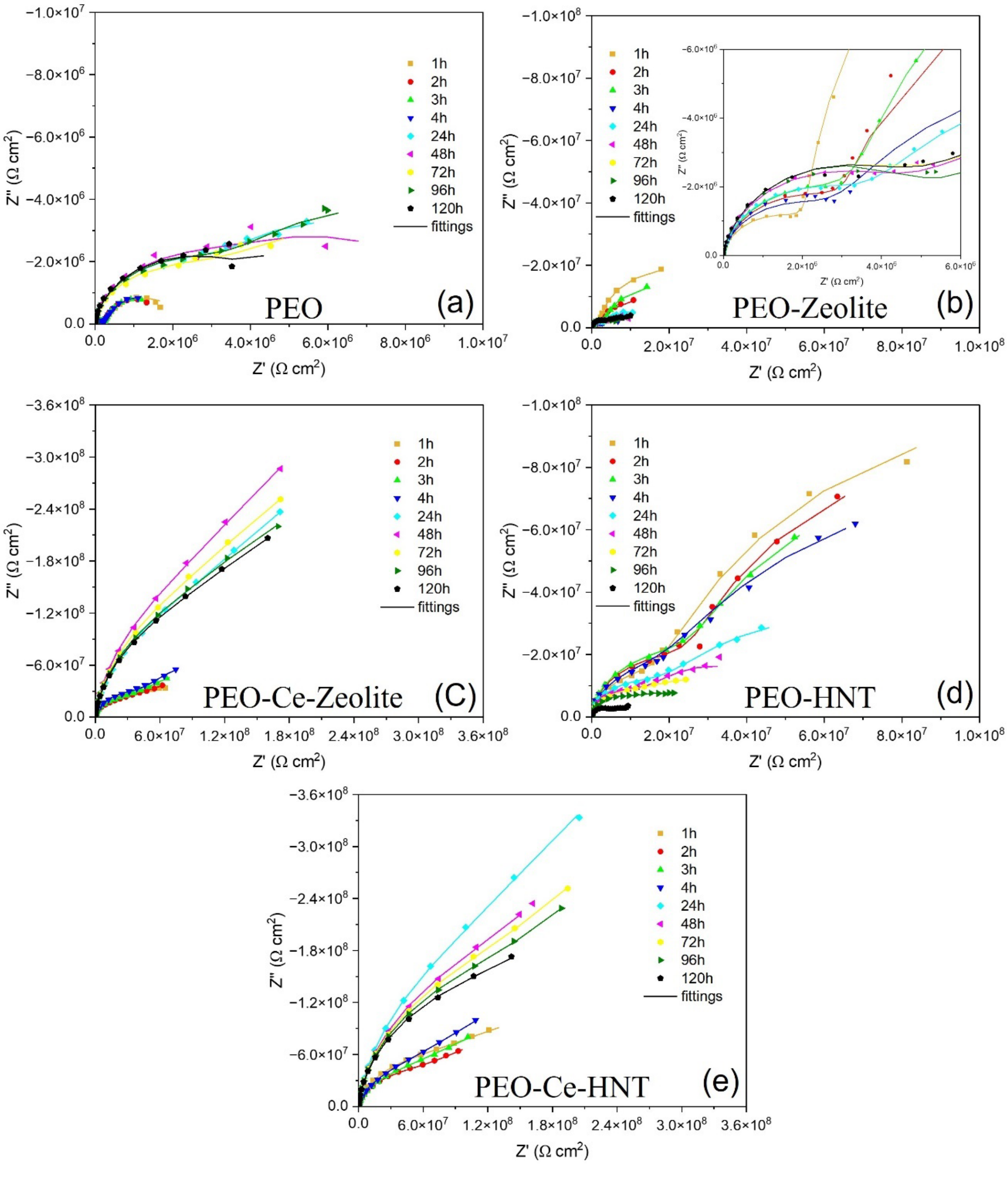

Supplementary Figure 2. EIS Nyquist plots of PEO (a), PEO-Zeolite (b), PEO-Ce-Zeolite (c), PEO-HNT (d), and PEO-Ce-HNT (e) after immersion in natural aerated 3.5 wt.% NaCl solution up to 120 h.

2.2 Electrochemical impedance spectroscopy fitting data

Supplementary Table 1. Electrical circuit fitting results for impedance spectra of PEO immersed in 3.5 wt. % NaCl solution for 120h.

Time (h)	CPE_1 ($S\ s^n\ cm^2$)	CPE-n	R_1 ($\Omega.cm^2$)	CPE_2 ($S\ s^n\ cm^2$)	CPE-n	R_2 ($\Omega.cm^2$)	CPE_3 ($S\ s^n\ cm^2$)	CPE-n	R_3 ($\Omega.cm^2$)
1	1.52×10^{-7}	0.87	1.69×10^{5}	4.09×10^{-6}	0.91	1.23×10^{6}			
2	2.38×10^{-7}	0.86	1.74×10^{5}	7.22×10^{-6}	0.96	1.34×10^{6}			
3	3.25×10^{-7}	0.85	2.06×10^{5}	8.18×10^{-6}	0.96	1.52×10^{6}			
4	4.04×10^{-7}	0.81	2.98×10^{5}	6.74×10^{-6}	0.91	2.01×10^{6}			
24	9.21×10^{-8}	0.97	272	2.32×10^{-7}	0.84	1.42×10^{6}	2.29×10^{-6}	0.93	1.06×10^{7}
48	5.46×10^{-8}	0.93	251	1.37×10^{-7}	0.88	4.88×10^{6}	1.50×10^{-6}	0.99	3.20×10^{6}
72	5.90×10^{-8}	0.99	264	1.36×10^{-7}	0.88	4.55×10^{6}	1.45×10^{-6}	0.97	5.12×10^{6}
96	6.09×10^{-8}	0.99	266	1.38×10^{-7}	0.88	4.49×10^{6}	1.42×10^{-6}	0.88	7.24×10^{6}
120	6.28×10^{-8}	0.99	260	1.44×10^{-7}	0.88	4.90×10^{6}	3.30×10^{-6}	0.99	5.08×10^{6}

Supplementary Table 2. Electrical circuit fitting results for impedance spectra of PEO-Zeolite immersed in 3.5 wt.% NaCl solution for 120h.

Time (h)	CPE_1 ($S\ s^n\ cm^2$)	CPE-n	R_1 ($\Omega.cm^2$)	CPE_2 ($S\ s^n\ cm^2$)	CPE-n	R_2 ($\Omega.cm^2$)	CPE_3 ($S\ s^n\ cm^2$)	CPE-n	R_3 ($\Omega.cm^2$)
1	5.68×10^{-8}	0.89	2.67×10^{6}	4.42×10^{-7}	0.99	3.78×10^{7}			
2	5.37×10^{-8}	0.89	4.01×10^{6}	4.28×10^{-7}	0.99	1.72×10^{7}			
3	5.33×10^{-8}	0.90	4.37×10^{6}	3.54×10^{-7}	0.94	2.85×10^{7}			
4	5.33×10^{-8}	0.90	3.28×10^{6}	5.02×10^{-7}	0.80	1.31×10^{7}			
24	5.66×10^{-8}	0.91	3.97×10^{4}	5.78×10^{-9}	0.90	4.21×10^{6}	6.12×10^{-7}	0.81	1.22×10^{7}
48	5.36×10^{-8}	0.92	8.48×10^{3}	1.31×10^{-8}	0.89	5.30×10^{6}	8.06×10^{-7}	0.81	7.96×10^{6}
72	5.09×10^{-8}	0.91	2.47×10^{3}	2.05×10^{-8}	0.90	5.82×10^{6}	9.01×10^{-7}	0.89	6.57×10^{6}
96	5.34×10^{-8}	0.91	2.43×10^{3}	2.26×10^{-8}	0.89	5.91×10^{6}	1.44×10^{-8}	0.87	5.94×10^{6}
120	5.60×10^{-8}	0.91	2.38×10^{3}	2.41×10^{-8}	0.89	6.29×10^{6}	1.34×10^{-6}	0.96	3.38×10^{6}

Supplementary Table 3. Electrical circuit fitting results for impedance spectra of PEO-HNT immersed in 3.5 wt.% NaCl solution for 120h.

Time (h)	CPE_1 ($S\ s^n\ cm^2$)	CPE-n	R_1 ($\Omega.cm^2$)	CPE_2 ($S\ s^n\ cm^2$)	CPE-n	R_2 ($\Omega.cm^2$)	CPE_3 ($S\ s^n\ cm^2$)	CPE-n	R_3 ($\Omega.cm^2$)
1	1.93×10^{-8}	0.96	2.65×10^{7}	7.24×10^{-8}	0.87	2.20×10^{8}			

2	1.99×10^{-8}	0.96	3.72×10^{7}	8.48×10^{-8}	0.92	1.87×10^{8}			
3	2.03×10^{-8}	0.96	4.11×10^{7}	8.61×10^{-8}	0.96	1.43×10^{8}			
4	2.04×10^{-8}	0.96	2.91×10^{7}	6.39×10^{-8}	0.80	1.73×10^{8}			
24	2.35×10^{-8}	0.95	1.49×10^{5}	1.22×10^{-9}	0.99	2.06×10^{7}	1.39×10^{-7}	0.73	8.75×10^{7}
48	2.79×10^{-8}	0.95	1.29×10^{5}	1.39×10^{-9}	0.99	1.74×10^{7}	2.04×10^{-7}	0.85	3.46×10^{7}
72	3.06×10^{-8}	0.94	6.24×10^{4}	2.12×10^{-9}	0.98	1.61×10^{7}	1.95×10^{-7}	0.76	2.72×10^{7}
96	3.33×10^{-8}	0.94	1.43×10^{4}	4.11×10^{-9}	0.93	1.37×10^{7}	3.44×10^{-7}	0.86	1.34×10^{7}
120	3.00×10^{-8}	0.95	5.77×10^{3}	1.61×10^{-9}	0.87	6.17×10^{7}	8.30×10^{-6}	0.87	5.51×10^{6}

Supplementary Table 4. Electrical circuit fitting results for impedance spectra of PEO-Ce-Zeolite immersed in 3.5 wt.% NaCl solution for 120h.

Time (h)	CPE_1 ($S\ s^n\ cm^2$)	CPE-n	R_1 ($\Omega.cm^2$)	CPE_2 ($S\ s^n\ cm^2$)	CPE-n	R_2 ($\Omega.cm^2$)	CPE_3 ($S\ s^n\ cm^2$)	CPE-n	R_3 ($\Omega.cm^2$)
1	1.80×10^{-8}	0.89	3.91×10^{4}	5.40×10^{-9}	0.92	5.23×10^{7}	1.25×10^{-7}	0.67	2.74×10^{8}
2	1.87×10^{-8}	0.90	2.75×10^{4}	6.12×10^{-9}	0.92	4.12×10^{7}	1.20×10^{-7}	0.73	1.05×10^{9}
3	1.92×10^{-8}	0.90	2.23×10^{4}	6.67×10^{-9}	0.91	4.82×10^{7}	1.39×10^{-7}	0.81	1.21×10^{8}
4	1.98×10^{-8}	0.91	2.16×10^{4}	6.65×10^{-9}	0.91	6.04×10^{7}	1.43×10^{-7}	0.87	1.40×10^{8}
24	2.09×10^{-8}	0.93	3.07×10^{4}	4.23×10^{-9}	0.95	3.32×10^{8}	5.50×10^{-8}	0.99	5.32×10^{8}
48	2.77×10^{-8}	0.93	4.17×10^{4}	3.11×10^{-9}	0.97	4.65×10^{8}	4.26×10^{-8}	0.99	7.52×10^{8}
72	2.43×10^{-8}	0.93	6.01×10^{4}	2.48×10^{-9}	0.97	3.86×10^{8}	5.29×10^{-8}	0.99	5.27×10^{8}
96	2.58×10^{-8}	0.93	7.32×10^{4}	2.20×10^{-9}	0.99	3.42×10^{8}	6.56×10^{-8}	0.99	3.90×10^{8}
120	2.70×10^{-8}	0.93	7.33×10^{4}	2.26×10^{-9}	0.99	3.22×10^{8}	7.33×10^{-8}	0.99	3.81×10^{8}

Supplementary Table 5. Electrical circuit fitting results for impedance spectra of PEO-Ce-HNT immersed in 3.5 wt.% NaCl solution for 120h.

Time (h)	CPE_1 ($S\ s^n\ cm^2$)	CPE-n	R_1 ($\Omega.cm^2$)	CPE_2 ($S\ s^n\ cm^2$)	CPE-n	R_2 ($\Omega.cm^2$)	CPE_3 ($S\ s^n\ cm^2$)	CPE-n	R_3 ($\Omega.cm^2$)
1	1.76×10^{-8}	0.92	3.18×10^{6}	2.42×10^{-9}	0.92	9.54×10^{7}	4.81×10^{-8}	0.57	3.65×10^{9}
2	1.92×10^{-8}	0.92	1.81×10^{6}	3.00×10^{-9}	0.83	6.13×10^{7}	4.95×10^{-8}	0.51	1.82×10^{9}
3	2.09×10^{-8}	0.91	3.66×10^{6}	1.28×10^{-9}	0.99	4.86×10^{7}	3.46×10^{-8}	0.50	8.55×10^{8}
4	2.13×10^{-8}	0.92	4.20×10^{6}	1.14×10^{-9}	0.99	6.11×10^{7}	3.25×10^{-8}	0.54	140×10^{9}
24	2.07×10^{-8}	0.93	3.43×10^{6}	1.07×10^{-9}	0.99	5.31×10^{8}	3.61×10^{-8}	0.99	8.58×10^{8}
48	2.10×10^{-8}	0.94	1.92×10^{6}	1.18×10^{-9}	0.99	4.17×10^{7}	5.44×10^{-8}	0.99	8.25×10^{8}
72	2.18×10^{-8}	0.94	1.69×10^{6}	1.29×10^{-9}	0.99	3.79×10^{8}	6.18×10^{-8}	0.99	6.53×10^{8}
96	2.25×10^{-8}	0.94	1.67×10^{6}	1.28×10^{-9}	0.99	3.53×10^{7}	7.44×10^{-8}	0.99	6.39×10^{8}
120	2.35×10^{-8}	0.94	1.68×10^{6}	1.25×10^{-9}	0.99	3.28×10^{7}	9.14×10^{-8}	0.99	6.79×10^{8}

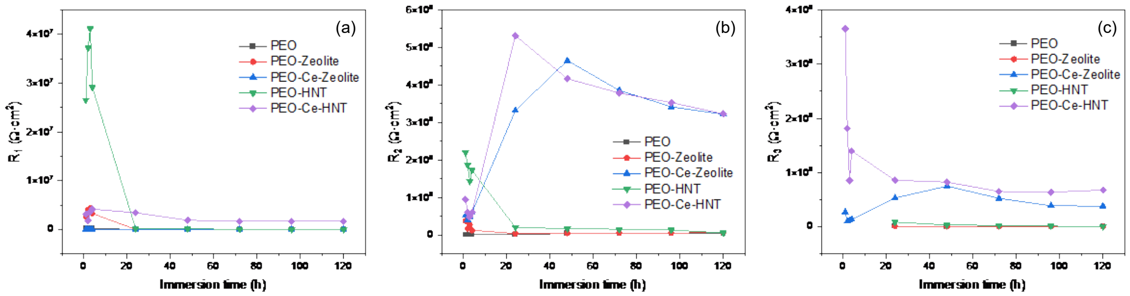

Supplementary Figure 3. Resistance parameters as a function of immersion time obtained from EIS graphs for different PEO coatings with nanocontainers.

2.3 Characterisation of the specimens after EIS testing

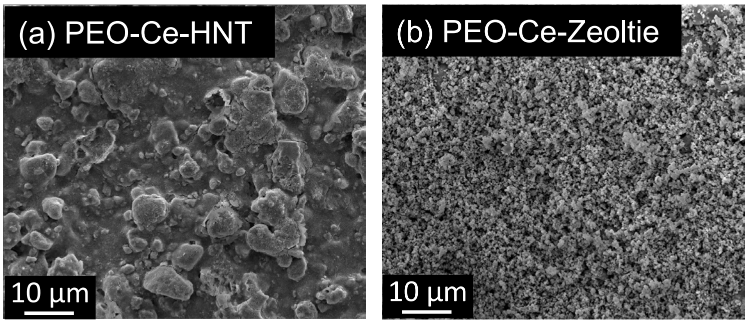

Supplementary Figure 4. SEM Micrographs of the surface of (a) PEO-Ce-HNT and (b) PEO-Ce-Zeolite after EIS 120 h immersion in naturally aerated 3.5 wt. % NaCl solution.

Supplementary References

1 in *Collection of Simulated XRD Powder Patterns for Zeolites* (eds M. M. J. Treacy, J. B. Higgins, & John B. Higgins) 373-379 (Elsevier Science B.V., 2001).

2 Dal Pozzo, D. M. *et al.* Free fatty acids esterification catalyzed by acid Faujasite type zeolite. *RSC Advances* **9**, 4900-4907 (2019). https://doi.org:10.1039/C8RA10248A

3 Wu, X. *et al.* Synthesis and adsorption properties of halloysite/carbon nanocomposites and halloysite-derived carbon nanotubes. *Applied Clay Science* **119**, 284-293 (2016). https://doi.org:https://doi.org/10.1016/j.clay.2015.10.029

4 Al Abri, S. *et al.* In-situ incorporation of Ce-zeolite during soft sparking plasma electrolytic oxidation. *Journal of Materials Research and Technology* **30**, 2365-2376 (2024). https://doi.org:https://doi.org/10.1016/j.jmrt.2024.04.011

5 Dias, S. A. S., Lamaka, S. V., Nogueira, C. A., Diamantino, T. C. & Ferreira, M. G. S. Sol–gel coatings modified with zeolite fillers for active corrosion protection of AA2024. *Corrosion Science* **62**, 153-162 (2012). https://doi.org:https://doi.org/10.1016/j.corsci.2012.05.009

6 Jiang, B.-L., Jiang, N. & Chang, Y.-X. Synthesis of highly active Cu(I)-Y(III)-Y zeolite and its selective adsorption desulfurization performance in presence of xylene isomers. *Petroleum Science* **18**, 295-306 (2021). https://doi.org:10.1007/s12182-020-00531-0

7 Li, X., Zhang, X. & Lei, L. Preparation of CuNaY zeolites with microwave irradiation and their application for removing thiophene from model fuel. *Separation and*

Purification Technology **64**, 326-331 (2009). https://doi.org:https://doi.org/10.1016/j.seppur.2008.10.016

8 Song, H. *et al.* Deep desulfurization of model gasoline by selective adsorption over Cu–Ce bimetal ion-exchanged Y zeolite. *Fuel Processing Technology* **116**, 52-62 (2013). https://doi.org:https://doi.org/10.1016/j.fuproc.2013.04.017

9 Adsul, S. H., Bagale, U. D., Sonawane, S. H. & Subasri, R. Release rate kinetics of corrosion inhibitor loaded halloysite nanotube-based anticorrosion coatings on magnesium alloy AZ91D. *Journal of Magnesium and Alloys* **9**, 202-215 (2021). https://doi.org:https://doi.org/10.1016/j.jma.2020.06.010

10 Lvov, Y. M., Shchukin, D. G., Möhwald, H. & Price, R. R. Halloysite Clay Nanotubes for Controlled Release of Protective Agents. *ACS Nano* **2**, 814-820 (2008). https://doi.org:10.1021/nn800259q

11 Pandey, G., Munguambe, D. M., Tharmavaram, M., Rawtani, D. & Agrawal, Y. K. Halloysite nanotubes - An efficient 'nano-support' for the immobilization of α-amylase. *Applied Clay Science* **136**, 184-191 (2017). https://doi.org:https://doi.org/10.1016/j.clay.2016.11.034

12 Alberola, J. A., Mondragón, R., Juliá, J. E., Hernández, L. & Cabedo, L. Characterization of halloysite-water nanofluid for heat transfer applications. *Applied Clay Science* **99**, 54-61 (2014). https://doi.org:https://doi.org/10.1016/j.clay.2014.06.012

13 Rawtani, D. *et al.* Development of a novel 'nanocarrier' system based on Halloysite Nanotubes to overcome the complexation of ciprofloxacin with iron: An in vitro approach. *Applied Clay Science* **150**, 293-302 (2017). https://doi.org:https://doi.org/10.1016/j.clay.2017.10.002

14 Rahul, R., Satyarthi, J. & Darbha, S. Lanthanum and zinc incorporated hydrotalcites as solid base catalysts for biodiesel and biolubricants production. *Indian Journal of Chemistry-Part A InorganicPhysical Theoretical and Analytical* **50**, 1017 (2011).
